# Supplementary material for: PhosIDP: a web tool to visualize the location of phosphorylation sites in disordered regions
Source: Sci Rep. 2021 May 11;11:9930. doi: 10.1038/s41598-021-88992-0 (PMC8113260; doi:10.1038/s41598-021-88992-0)

Supplementary Information

PhosIDP: a web tool to visualize the location of phosphorylation sites in disordered regions

Sonia T. Nicolaou[1,2], Max Hebditch[1], Owen J. Jonathan[1], Chandra S. Verma[2,3,4], Jim Warwicker[1,*]

[1]School of Biological Sciences, Faculty of Biology, Medicine and Health, Manchester Institute of Biotechnology, University of Manchester, Manchester M1 7DN, UK
[2]Bioinformatics Institute, Agency for Science, Technology, and Research (A*STAR), Singapore 138671, Singapore [3]School of Biological Sciences, Nanyang Technological University, 60 Nanyang Drive, Singapore 637551 [4]Department of Biological Sciences, National University of Singapore, 14 Science Drive 4, Singapore 117543

* jim.warwicker@manchester.ac.uk

Supplementary Figure 1

Variation of window size for charge and fold propensity plots for Q14011. Sliding windows of the charge and fold propensity plots were changed to 5, 10 and 40, and displayed in comparison with the 21 amino acid window results.

Supplementary Figure 1

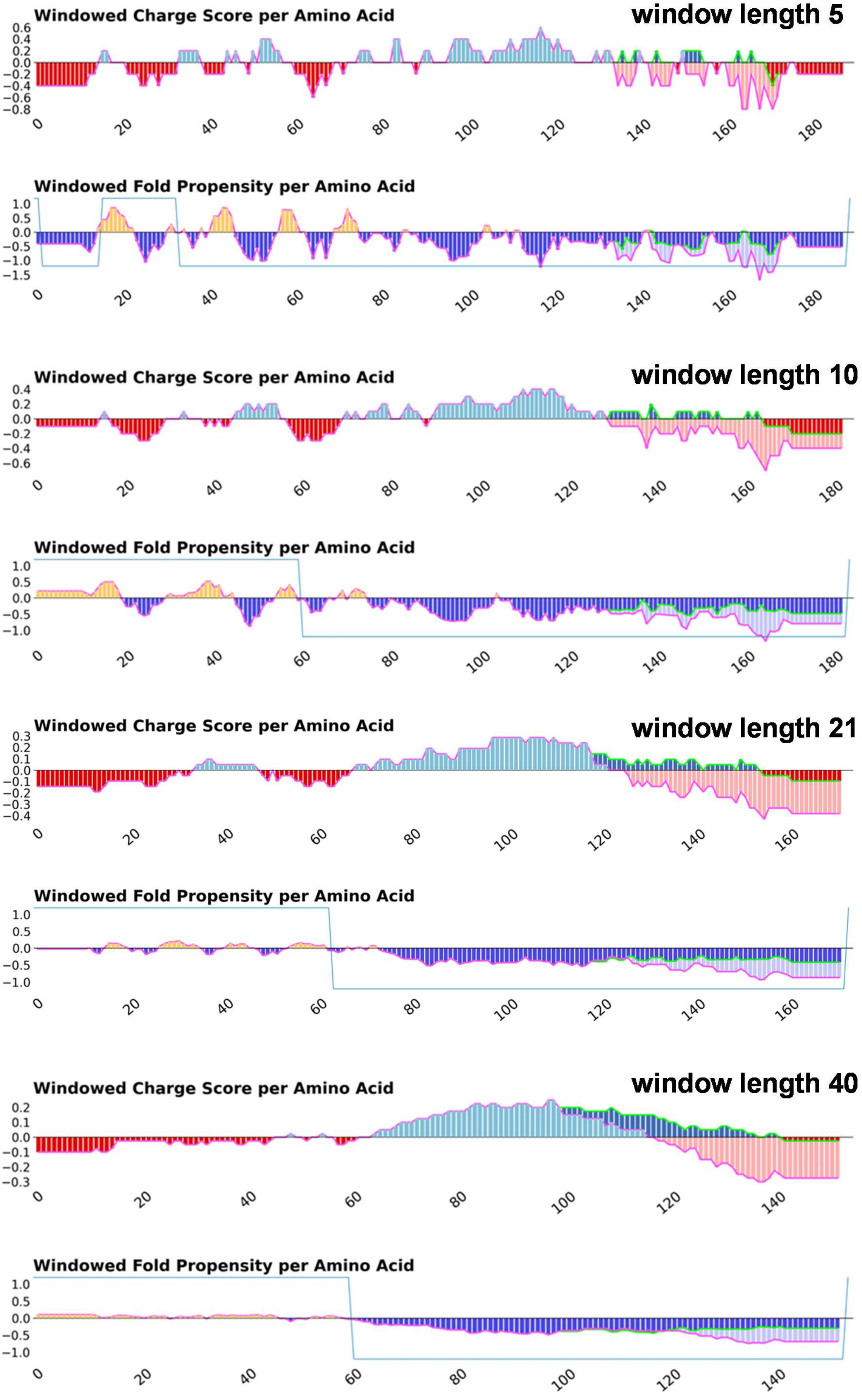

Supplementary Figure 2

Comparison of disorder prediction schemes, for CIRBP (Q14011) and nucleophosmin (P06748). Results from 4 predictors are shown, fold propensity as used in the phosIDP server, IUPred2-long, MetaDisorderMD, and PDisorder (see main text). Note that phosIDP and PDisorder predict disorder as negative, and IUPred2-Long and MetaDisorderMD predict disorder as positive, indicated with labels of IDR (disorder) and STR (ordered, structured).

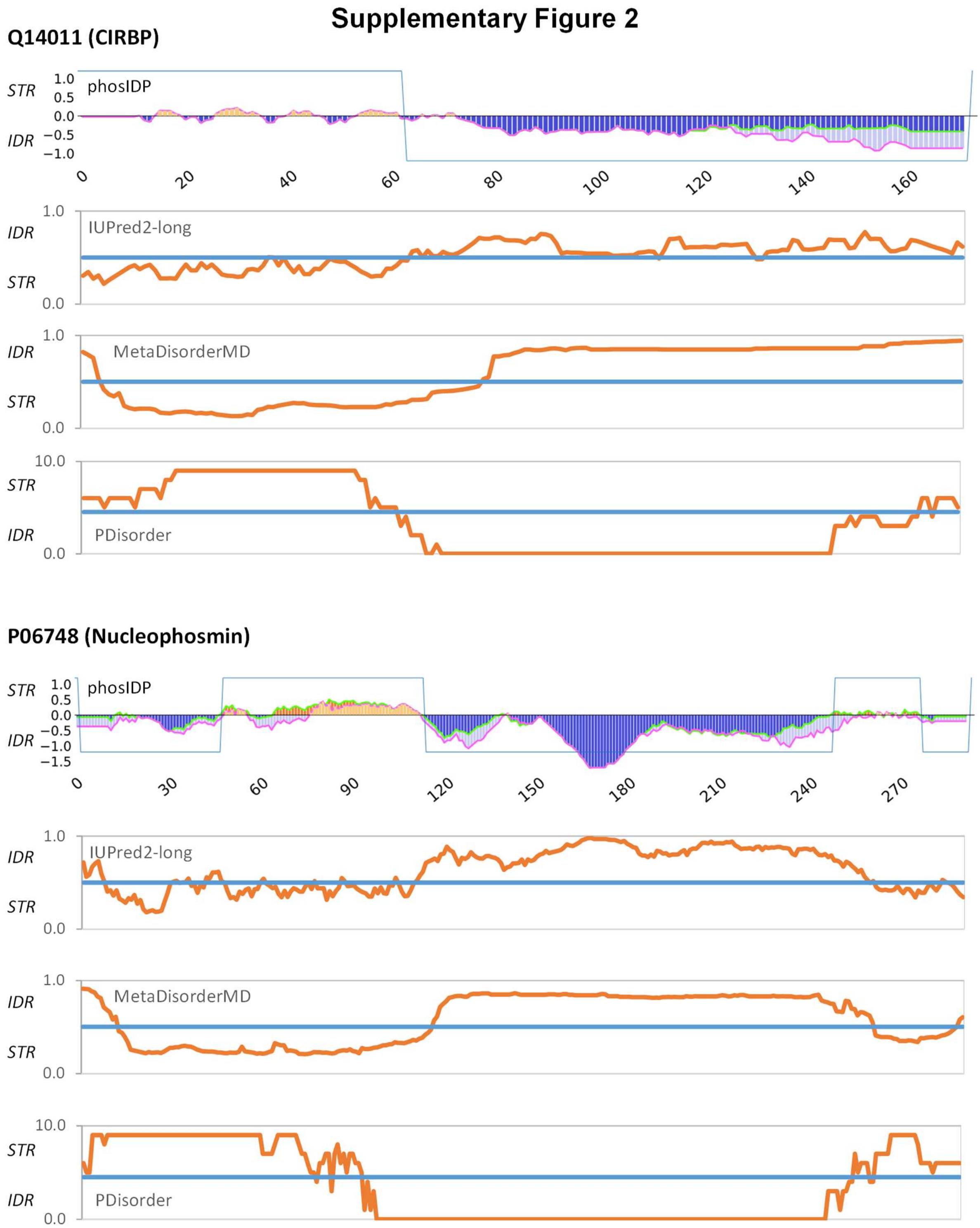

Supplement: Supplementary file 1 — Supplementary Information [file 41598_2021_88992_MOESM1_ESM.pdf]
